# Thermally stable Ni foam-supported inverse CeAlO_x_/Ni ensemble as an active structured catalyst for CO_2_ hydrogenation to methane

**DOI:** 10.1038/s41467-024-47403-4

**Published:** 2024-04-10

**Authors:** Xin Tang, Chuqiao Song, Haibo Li, Wenyu Liu, Xinyu Hu, Qiaoli Chen, Hanfeng Lu, Siyu Yao, Xiao-nian Li, Lili Lin

**Affiliations:** 1 Institute of Industrial Catalysis, State Key Laboratory of Green Chemistry Synthesis Technology, College of Chemical Engineering, Zhejiang University of Technology, Hangzhou, Zhejiang 310014 China; 2 Zhejiang Carbon Neutral Innovation Institute & Zhejiang International Cooperation Base for Science and Technology on Carbon Emission Reduction and Monitoring, Zhejiang University of Technology, Hangzhou, 310014 China; 3 Key Laboratory of Biomass Chemical Engineering of Ministry of Education, College of Chemical and Biological Engineering, Zhejiang University Hangzhou, 310027 China

**Keywords:** Heterogeneous catalysis, Catalyst synthesis, Materials for energy and catalysis

## Abstract

Nickel is the most widely used inexpensive active metal center of the heterogeneous catalysts for CO_2_ hydrogenation to methane. However, Ni-based catalysts suffer from severe deactivation in CO_2_ methanation reaction due to the irreversible sintering and coke deposition caused by the inevitable localized hotspots generated during the vigorously exothermic reaction. Herein, we demonstrate the inverse CeAlO_x_/Ni composite constructed on the Ni-foam structure support realizes remarkable CO_2_ methanation catalytic activity and stability in a wide operation temperature range from 240 to 600 °C. Significantly, CeAlO_x_/Ni/Ni-foam catalyst maintains its initial activity after seven drastic heating-cooling cycles from RT to 240 to 600 °C. Meanwhile, the structure catalyst also shows water resistance and long-term stability under reaction condition. The promising thermal stability and water-resistance of CeAlO_x_/Ni/Ni-foam originate from the excellent heat and mass transport efficiency which eliminates local hotspots and the formation of Ni-foam stabilized CeAlO_x_/Ni inverse composites which effectively anchored the active species and prevents carbon deposition from CH_4_ decomposition.

## Abstract (web summary)

An inverse CeAlO_x_/Ni/Ni-foam structured catalyst with high thermal stability and water resistance is shown to be effective at CO_2_ methanation across a wide temperature range because of efficient heat/mass transport.

## Introduction

Ni-based catalysts are widely applied in the industrial CO methanation reaction and have shown great potential for the conversion of CO_2_ to CH_4_ (also known as the Sabatier reaction), due to the relatively high activity, selectivity and affordability^1–4^. Considering the potential application of CO_2_ methanation in the integrated power-to-gas process containing CO_2_ capture, renewable energy-powered hydrogen production (e.g., electrolysis of water) and CO_2_ utilization modules, the development of active and durable Ni-based CO_2_ methanation catalyst is highly desirable and urgently demanded^5–7^.

Despite the promising perspective, it is challenging to apply conventional Ni/oxide catalysts in CO_2_ methanation reactions. One of the problems is the formation of localized hotspots in the catalyst bed caused by the severe reaction heat and the relatively poor heat conductivity of oxide hosting materials^8–10^. According to the literature estimation, an adiabatic temperature rise around 59.2 °C will be presented for each 1 mol% conversion of CO_2_ in the hydrogenation reaction, and the adiabatic temperature can reach the maxima of 600 °C, the temperature corresponding to the thermal balance between exothermic CO_2_ methanation and endothermic reverse water gas shift reactions^11,12^. Once the thermal disturbance exceeds the binding energy between Ni nanoparticle and support, the well-dispersed Ni species tend to migrate on the catalyst and further agglomerate into large particles driven by the surface energy^13,14^. What’s more, the high concentration of steam in the product of CO_2_ hydrogenation aggravates the sintering of Ni species. Thus, the rational design of anti-sintering and water-resistant Ni-based catalysts is demanding to overcome the stability challenges in the CO_2_ hydrogenation to methane^15^.

Structured metal materials like Ni-foam and Cu-foam etc. with high heat conductivity, rich channels and mechanical robustness provide opportunities to eliminate the undesirable generation of local hotspots^16–20^. However, the applications of metal-structured catalysts are limited due to the unfavorable catalytic functionalization of active sites (Ni/oxide) and poor adherence of metal oxides on the surface of metal foam skeleton (Ni/oxide/Ni-foam)^21–25^. Regarding the existing challenges and inspired by our previous studies on the inverse catalysts with improved CO_2_ hydrogenation performances, we propose the construction of nano-oxide/Ni inverse structure on Ni-foam as the active site for CO_2_ methanation in order to exploit the advantages of structured and inverse catalysts^26–32^. Particularly, by growing a closely contact layer of nickel hydroxide on the Ni-foam substrate via an etching process as the attaching sites of nano-oxides, a fine and uniform dispersion of oxide/NiO nano-composites over Ni foam with high density and strong structure robustness can be obtained as the precursor of oxide/Ni inverse structure^33–35^. The inverse oxide/Ni active species functionalized Ni foam structured catalyst will simultaneously enhance the CO_2_ hydrogenation activities and realized remarkable stability.

In this work, we report a Ni-foam supported inverse CeAlO_x_/Ni species (CeAlO_x_/Ni/Ni-foam) as an efficient structured catalyst for CO_2_ hydrogenation towards methane. The inverse CeAlO_x_/Ni/Ni-foam catalyst presents significantly improved methane productivity at low temperature and exhibits superior thermal stability, and its activity remains virtually unchanged after seven cycles of heating-cooling treatment (25–600 °C) and 200 h time-on-stream (at 240 °C) without significant sintering or carbon deposition. The structured catalyst also shows excellent water resistance, and the CO_2_ methanation activity can be reversibly recovered after the removal of excessive steam. Besides the excellent stability, the structured catalyst also realizes a CO_2_ conversion above 80% at 240 °C with a CH_4_ selectivity over 98.6% at GHSV of 80,000 h^−1^, 14 times higher than the conventional Ni/oxide references. This design and fabrication of the structured catalyst with inverse species as active sites provide a general strategy and a promising platform to construct high-performance and durable catalysts for CO_2_ hydrogenation reaction to methane.

## Result

### Structural characterization of catalysts

**Fig. 1:**
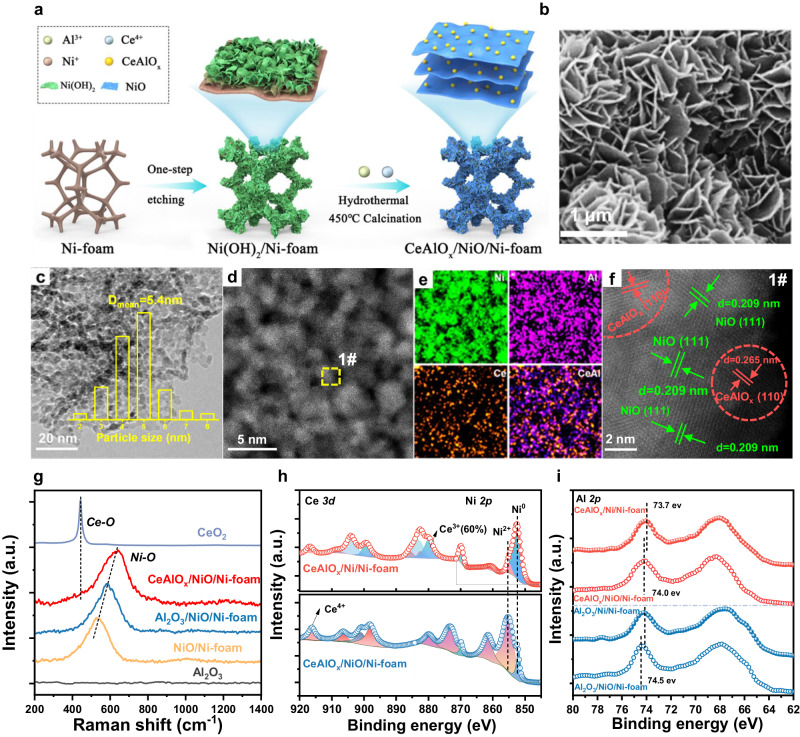
Catalyst preparation strategy and structure characterization. **a** Schematic diagram of the synthesis of CeAlO_x_/NiO/Ni-foam catalyst; **b** SEM image of CeAlO_x_/Ni(OH)_2_/Ni-foam catalyst; **c** TEM image of CeAlO_x_/NiO catalyst scraped from the Ni-foam substrate (inset is the particle size distribution histogram); **d** Aberration-corrected HAADF-STEM image of scraped CeAlO_x_/NiO catalyst; **e** EDS elemental maps of scraped CeAlO_x_/NiO catalyst, showing the distribution of Ni, Ce and Al; **f** High-resolution HAADF-STEM image of the 1# area in **d**; **g** Raman spectra of the NiO/Ni-foam, Al_2_O_3_/NiO/Ni-foam, CeAlO_x_/NiO/Ni-foam, CeO_2_ and Al_2_O_3_ catalysts; **h** In situ XPS of Ce 3*d* and Ni 2*p* of CeAlO_x_/NiO/Ni-foam and CeAlO_x_/Ni/Ni-foam catalysts; **i** In situ XPS of Al 2*p* of CeAlO_x_/NiO/Ni-foam, CeAlO_x_/Ni/Ni-foam, Al_2_O_3_/NiO/Ni-foam and Al_2_O_3_/Ni/Ni-foam catalysts.

The Ni(OH)_2_ overlayer-covered Ni-foam is prepared using a urea hydrothermal etching method. The following modification of the Ni(OH)_2_ layer with Ce and Al oxides is realized by hydrothermal method followed by calcination at 400 °C (Fig. 1a). The prepared catalyst is labeled as CeAlO_x_/NiO/Ni-foam, and the loadings of Al and Ce are 2.5 wt.% and 2.4 wt.% (about 10.4 wt.% and 11.2 wt.% respective to NiO overlayer, determined by inductively coupled plasma-optical emission spectrometer (ICP-OES)). Other reference catalysts including the Al_2_O_3_/NiO/Ni-foam and NiO/Ni-foam are prepared with the same procedure. Before performance evaluation, all catalysts are pre-reduced in 20% H_2_ at 450 °C for 3 h to convert the NiO substrate into metallic Ni to generate the inverse oxide/Ni composites on Ni foam skeleton (labeled as CeAlO_x_/Ni/Ni-foam, Al_2_O_3_/Ni/Ni-foam and Ni/Ni-foam). Ni supported on the Al_2_O_3_ and CeAlO_x_ oxide supports are prepared by the precipitation method (Ni loading is controlled at 13 wt%) to compare with the inverse oxide/Ni composite catalysts, which helps to understand the importance of Ce doping.

X-ray diffraction (XRD) (Supplementary Fig. 1) patterns of the CeAlO_x_/NiO/Ni-foam, Al_2_O_3_/NiO/Ni-foam and NiO/Ni-foam show intense diffraction peaks corresponding to NiO and Ni-foam substrate. After modification with Ce and Al oxides, broad peaks at 20°~25° appear in the CeAlO_x_/NiO/Ni-foam and Al_2_O_3_/NiO/Ni-foam, which suggests fine dispersion of Ce and Al oxide species on the substrate due to the anchoring of the Ni(OH)_2_ overlayer^36,37^. All catalysts exhibit type-IV isotherms with type-H3 hysteresis loops, indicating the presence of mesopores (Supplementary Fig. 2) and the Barrett-Joyner-Halenda apertures in the structured catalysts^38^. The average pore sizes of CeAlO_x_/NiO/Ni-foam, Al_2_O_3_/NiO/Ni-foam, and NiO/Ni-foam catalysts are ~6 nm, 5.5 nm, and 4.3 nm (Supplementary Table 1).

The morphology and microstructure of CeAlO_x_/NiO/Ni-foam are further observed using electron microscopic methods. The scanning electron microscopy (SEM) images of CeAlO_x_/NiO/Ni-foam preserves monolith geometry and rich 3-dimensional cross-connected pore structure after the modification and thermal treatments (Supplementary Fig. 3). Th CeAlO_x_/NiO composite displays a honeycomb-like nanoflake appearance on the skeleton of Ni foam with an average thickness of 4 µm (Fig. 1b and Supplementary Fig. 3). The adherence of CeAlO_x_/NiO on Ni foam is sufficiently strong to bare the vigorous ultrasonic treatment (Supplementary Fig. 5), which highlights the effectiveness of the NiO overlayer in anchoring the fine oxide species. Transmission electron microscopy (TEM) images of CeAlO_x_/NiO sample scraped from the structured catalysts suggest that NiO nanoparticles (distribution centered at ~5.4 nm) are deposited on the exterior surface of Ni-foam (Fig. 1c and Supplementary Fig. 4). High angle dark field scanning transmission electron microscopy (HAADF-STEM) and energy-dispersive X-ray (EDS) element mapping images further confirm the uniform dispersion of Ce and Al over NiO particles in the nano-composite (Fig. 1d, e). Atomic-level image of region 1# from Fig. 1d shows the lattice fringes of 0.265 nm (correspond to CeAlO_3_(110), Supplementary Fig. 6), demonstrating the formation of CeAlO_x_ mixed oxide and loaded on the NiO support (Fig. 1f). After reduction, an inverse interface composed with CeAlO_x_ oxide particles on Ni support will be formed. Raman spectroscopy is performed to investigate the metal-O vibration of different Ni-foam structured catalyst (Fig. 1g). The peak at 540 ~ 650 cm^−1^ is confirmed to the contribution of Ni-O based on the comparison of passivated MO_x_/NiO/Ni-foam and reduced MO_x_/Ni/Ni-foam catalyst, as the Ni-O vibration peak disappears completely (Supplementary Fig. 7) due to the fully reduction of NiO to metallic Ni^39^. The redshift of Ni-O vibration peaks in the Al_2_O_3_/NiO/Ni-foam (580 cm^−1^) compares to that of NiO/Ni-foam (540 cm^−1^), which is probably the effect of the formation of Al-O-Ni coordination. Then, a larger red shift of Ni-O vibration appears when Ce is introduced to the Al_2_O_3_/NiO/Ni-foam inverse catalyst, and no Ce-O vibration is emerged, suggesting the formation of CeAlO_x_ mixed oxide which affects the Ni-O vibration^40^.

The chemical state of the catalyst surface is further explored by in situ X-ray photoelectron spectroscopy (XPS, peak fitting results in Supplementary Fig. 8 and Supplementary Table 2). From Ni 2*p* XPS spectra (Fig. 1h), it is confirmed that the surface of calcined CeAlO_x_/NiO/Ni-foam mainly corresponds to Ni^2+^ species (>76%), which converts into metallic Ni^0^ after reduction^41,42^. The Ce 3*d* (Fig. 1h) spectra show that over 60% surface Ce atoms become to Ce^3+^ species after reduction, which could introduce abundant oxygen vacancies in the inverse composite. Meanwhile, the 0.8 eV negative shift of the Al 2*p* XPS peak of the CeAlO_x_/Ni/Ni-foam sample compared with Al_2_O_3_/Ni/Ni-foam sample demonstrates the formation of CeAlO_x_ mixed metal oxides in the catalyst (Fig. 1i)^39,43^. The O_surface_/(O_surface_+O_lattice_) ratio of CeAlO_x_/Ni/Ni-foam catalyst reaches ~30% (based on the O 1 *s* region XPS spectra in Supplementary Fig. 6), which is in good agreement with the CeAlO_x_ mixed metal oxides contains higher density of oxygen vacancies based on O_2_-pulse chemisorption results (Supplementary Fig. 9).

### Catalytic performance of the structured catalysts

**Fig. 2:**
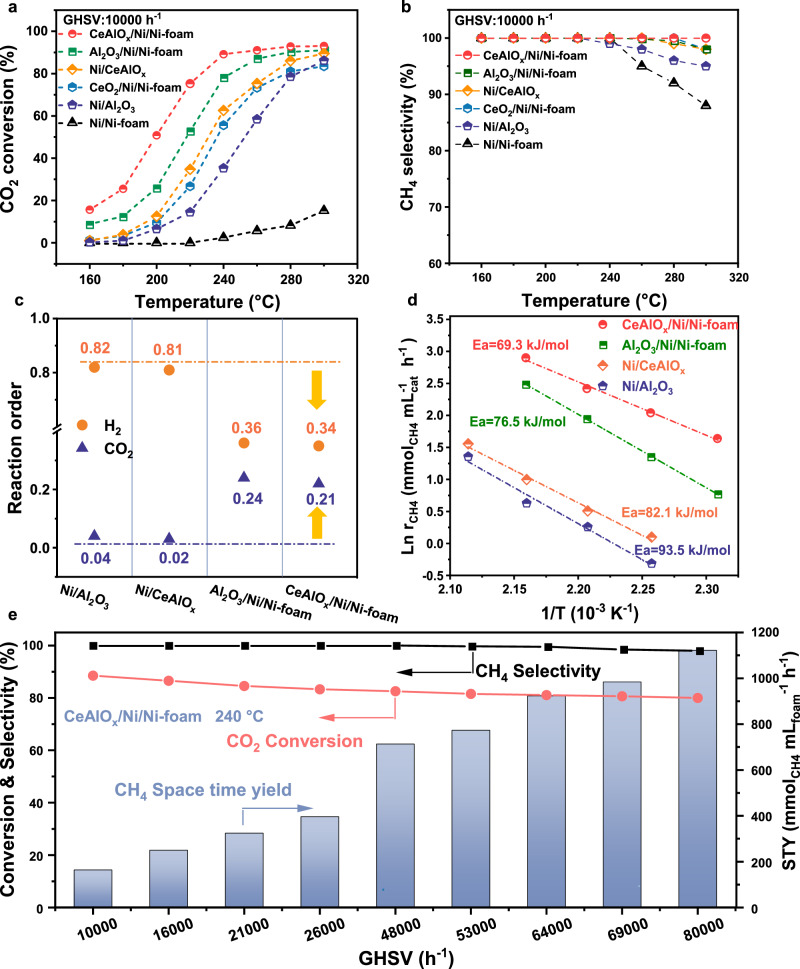
The catalytic performance of CeAlO_x_/Ni/Ni-foam catalyst. Temperature-dependent **a** CO_2_ conversion and **b** CH_4_ selectivity of the CeAlO_x_/Ni/Ni-foam, Al_2_O_3_/Ni/Ni-foam, CeO_2_/Ni/Ni-foam, Ni/Al_2_O_3_, and Ni/Ni-foam catalysts (reaction conditions: GHSV = 10,000 h^−1^, 160–300 °C CO_2_:H_2_:N_2_ = 18:72:10, *P* = 0.1 MPa); **c** Reaction orders with respect to H_2_ and CO_2_ for methane formation; **d** CH_4_ based apparent activation energy (*E*_*a*_) of CeAlO_x_/Ni/Ni-foam, Al_2_O_3_/Ni/Ni-foam, Ni/Al_2_O_3_ and Ni/CeAlO_x_ catalysts; **e** GHSV-dependent activities of CeAlO_x_/Ni/Ni-foam catalyst at 240 °C.

The catalytic performances of the structured catalysts for CH_4_ synthesis from CO_2_ hydrogenation are evaluated between 160–300 °C using a gas feed of CO_2_/H_2_/N_2_ = 18/72/10 under atmospheric pressure and a gas hourly space velocity (GHSV) of 10,000 h^−1^. The activities of MO_x_/Ni/Ni-foam (M = Y, Zr, Al, Ce, and Mg) catalysts and the Ni/Ni-foam catalyst in CO_2_ methanation reaction are showed in Fig. 2a, b and Supplementary Fig. 10. Almost no CO_2_ conversion is observed over the Ni/Ni-foam and Ni-foam substrates (below 250 °C). In comparison, 40–80% CO_2_ conversion are obtained at 250 °C on the MO_x_/Ni/Ni-foam catalysts, suggesting the importance of oxide modification in promoting the CO_2_ methanation activity. Furthermore, it is found the formation of Ce-Al mixed oxide phase (CeAlO_x_/Ni/Ni-foam catalyst) doubles the CO_2_ conversion at 200 °C compared with Al_2_O_3_/Ni/Ni-foam (Fig. 2a, b and Supplementary Fig. 11) and Ce/Al/Ni=1/5/30 is determined as the optimal composition. In the performance evaluation, CeAlO_x_/Ni/Ni-foam catalyst achieves ~90% CO_2_ conversion and CH_4_ selectivity of >99.9% at 240 °C, which far exceeds the conventional oxide-supported Ni catalysts. The space-time yields (STY) of CH_4_ of CeAlO_x_/Ni/Ni-Foam and corresponding Ni/CeAlO_x_ catalyst in kinetic region (CO_2_ conversion<15%^44^, Supplementary Fig. 12 and Supplementary Table 4) show that the CH_4_-STY of CeAlO_x_/Ni/Ni-foam catalyst is 65.3 mmol_CH4_/mL_foam_/h, which is 15 times higher than that of Ni/CeAlO_x_ catalyst.

For the comparison of CO_2_ and H_2_ reaction order, diluting CO_2_ reaction gas was applied to ensure that CO_2_ is converted in the kinetic region and the effect of hotspots is eliminated. Kinetic analysis of the CO_2_ methanation catalysts shows the apparent H_2_ and CO_2_ reaction orders of CeAlO_x_/Ni/Ni-foam are 0.34 and 0.21, and those of Al_2_O_3_/Ni/Ni-foam are 0.36 and 0.24. In comparison, the reaction orders of conventional Ni/CeAlO_x_ and Ni/Al_2_O_3_ are 0.81/0.02 and 0.82/0.04 (Fig. 2c and Supplementary Table 5). The change of the apparent kinetic orders of H_2_ and CO_2_ suggests that the CO_2_ coverage decreases and H_2_ surface coverage is intensified over the MO_x_ ensembles of CeAlO_x_/Ni/Ni-foam and Al_2_O_3_/Ni/Ni-foam according to the Langmuir-Hinshelwood mechanism, which could significantly promote the surface reaction. By varying the GHSV for different catalysts, it is ensured that all the CO_2_ conversion used to calculate *E*_*a*_ are below 6% (Supplementary Fig. 13). The CH_4_ base *E*_*a*_ of CeAlO_x_/Ni/Ni-foam is determined as 61.3 kJ/mol, lower than Al_2_O_3_/Ni/Ni-foam (76.5 kJ/mol) and much lower than that of conventional Ni/CeAlO_x_ (82.1 kJ/mol) and Ni/Al_2_O_3_ (93.5 kJ/mol), confirming the immense contribution of CeAlO_x_/Ni inverse structure on promotion of reaction kinetics in methane synthesis from CO_2_ hydrogenation reaction (Fig. 2d).

The STY of CH_4_ as a function of GHSV at 240 °C is further evaluated (Fig. 2e), and it is found that the CO_2_ conversion of CeAlO_x_/Ni/Ni-foam remains above 80% when the GHSV increases to 80,000 h^−1^. The corresponding STY of methane reaches 1109 mmol_CH4_/mL_foam_/h (4450 mmol/g_cat_/h with respect to the mass of CeAlO_x_/Ni ensemble), which is more competitive than the state-of-the-art supported Ru and Ni catalysts for the low-temperature CO_2_ methanation (Supplementary Table 6).

**Fig. 3:**
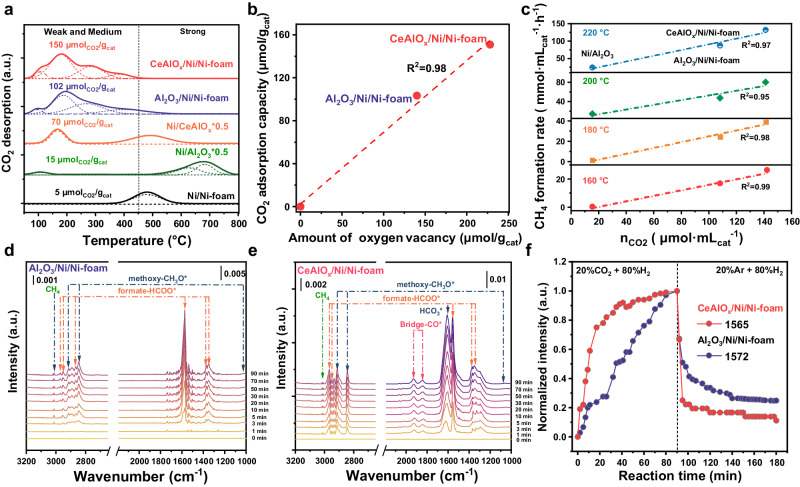
Investigation on the reaction mechanism of CeAlO_x_/Ni/Ni-foam catalyst. **a** The CO_2_-TPD profiles of Ni-base catalysts; **b** relationship between CO_2_ capture capacity and amount of oxygen vacancies on CeAlO_x_/Ni/Ni-foam and Al_2_O_3_/Ni/Ni-foam catalysts; **c** the correlation of the STY methanol and the amount of adsorbed CO_2_ at 50–400 °C; **d**, **e** DRIFTs results of t the Al_2_O_3_/Ni/Ni-foam catalyst and CeAlO_x_/Ni/Ni-foam catalyst in the stream of CO_2_/H_2_ mixture under 0.1 MPa respectively at 180 °C; **f** normalized intensities of the typical formate surface species as a function of reaction time (~1565 cm^−1^ for CeAlO_x_/Ni/Ni-foam; ~1572 cm^−1^ for Al_2_O_3_/Ni/Ni-foam).

To understand the excellent catalytic performance of the CeAlO_x_/Ni/Ni-foam structured catalyst, a number of characterizations are performed to identify the active sites. CO_2_ temperature program desorption profiles (Fig. 3a) show the amount of CO_2_ adsorbed at weak and medium alkaline sites are 150 and 102 μmol/g_cat_ (Supplementary Table 3). It can be seen that the capacity of weak- and medium- adsorbed CO_2_ display a near linear correlation with the density of oxygen vacancies (*R*^2^ = 0.98) (Fig. 3b). As the weak- and medium- adsorbed CO_2_ are determined to show a linear relationship with the intrinsic productivity of CH_4_ at 160, 180, 200, and 220 °C (Fig. 3c), it can be confirmed that the oxygen vacancies at the inverse oxide-metal interface are probably the sites for CO_2_ activation at low temperature, which accounts for the activity of CO_2_ methanation.

In situ diffuse reflectance Fourier transform infrared spectroscopy (DRIFTs) studies further elucidate that the structure of the MO_x_/Ni/Ni-foam composites affect the types and conversion rate of surface intermediates (Fig. 3d, e and Supplementary Fig. 14). Under the reaction atmosphere (CO_2_ + 4H_2_), bridged CO* (1833 and 1930 cm^−1^), formate (2970, 1563, 1380 cm^−1^) and methoxy (2845, 2926 cm^−1^) species are observed on CeAlO_x_/Ni/Ni-foam catalyst^45^. In contrast, only formate and methoxy species are observed on Al_2_O_3_/Ni/Ni-foam catalyst (Fig. 3d). In addition, when CO_2_ is removed from the feed after steady state is reached, CO* and formate species on the CeAlO_x_/Ni/Ni-foam catalyst are rapidly consumed together with the formation of methane, and the consumption of formate species and formation of methane is also observed on the Al_2_O_3_/Ni/Ni-foam (Fig. 3d and Supplementary Fig. 14), which indicates that both formate and CO* are important intermediates on the CeAlO_x_/Ni/Ni-foam catalyst, while methanation on the Al_2_O_3_/Ni catalyst mainly follows the formate pathway. Therefore, these two possible reaction pathways synergistically promote the lower temperature methanation on the CeAlO_x_/Ni/Ni-foam catalyst.

### Mechanism studies

**Fig. 4:**
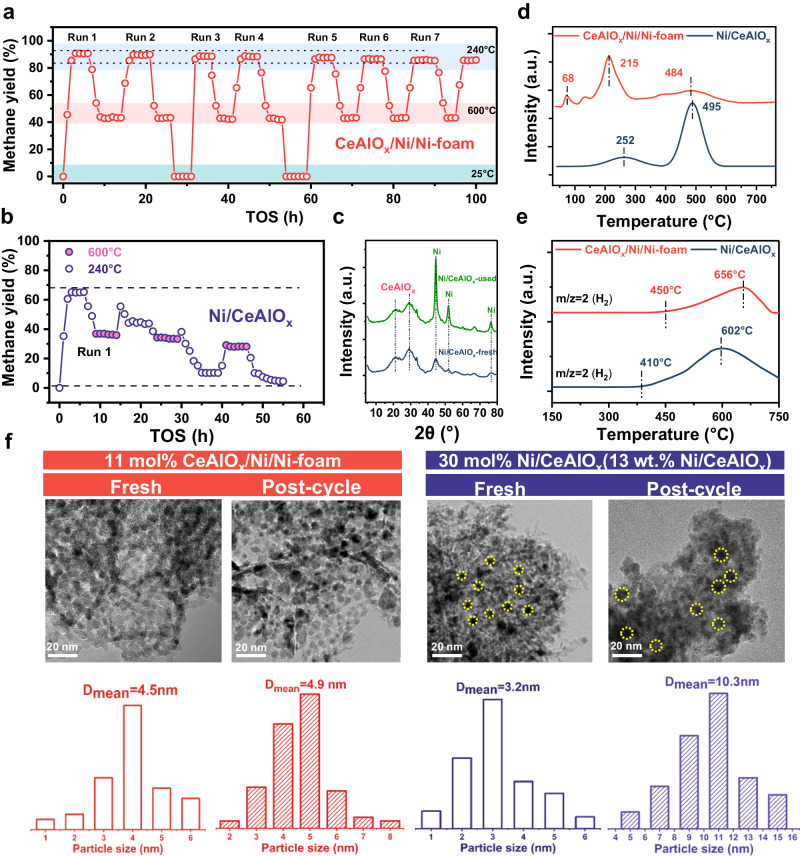
Thermal shock resistance of CeAlO_x_/Ni/Ni-foam catalyst. CH_4_ yield of **a** CeAlO_x_/Ni/Ni-foam and **b** Ni/CeAlO_x_ catalysts during heating-cooling treatment (reaction conditions: GHSV = 10,000 h^−1^, CO_2_:H_2_:N_2_ = 18:72:10, *P* = 0.1 MPa); **c** XRD spectra of Ni/CeAlO_x_ catalyst before and after cyclic reaction; **d** TPO results of CeAlO_x_/Ni/Ni-foam and Ni/CeAlO_x_ catalysts after heating-cooling cycle tests; **e** TPSR results of methane on Ni/CeAlO_x_ and CeAlO_x_/Ni/Ni-foam. Reaction conditions: 10 vol% CH_4_/Ar, GHSV = 15,000 h^−1^; **f** STEM images of CeAlO_x_/Ni/Ni-foam and Ni/CeAlO_x_ catalysts before and after heating-cooling cycle tests.

The reaction stability is probably one of the most important indicators for a practical catalyst, especially for the CO_2_ methanation catalyst, which faces significant challenges of sintering and carbon deposition^46^. To investigate the thermal shock resistance of CeAlO_x_/Ni/Ni-foam structure catalyst, a seven-cycle reciprocating heating-cooling test between 25 and 600 °C was performed (Fig. 4a). After each cycle, the CO_2_ conversion and CH_4_ selectivity of CeAlO_x_/Ni/Ni-foam at 240 °C can be restored (Fig. 4a). In contrast, the conventional Ni/CeAlO_x_ shows a rapid deactivation after only one heating-cooling cycle (Fig. 4b), which is probably due to the agglomeration of Ni NPs (see XRD patterns of the fresh and spent catalysts in Fig. 4c). This phenomenon implies that the interaction between the oxide and Ni substrate effectively inhibit the migration of Ni species and thereby prevent the undesirable sintering^47,48^. Additionally, the temperature program oxidation (TPO) experiment of the spent CeAlO_x_/Ni/Ni-foam and Ni/CeAlO_x_ catalysts also confirms coke formed on CeAlO_x_/Ni/Ni-foam after seven cycles is mainly amorphous carbon which can be oxidized around 215 °C. While large amount of partial crystalized carbon is generated on Ni/CeAlO_x_ after three cycles (mainly oxidized at 400~550 °C), demonstrating CeAlO_x_/Ni/Ni-foam structured catalyst is able to inhibit the formation of coke in CO_2_ methanation reaction (Fig. 4d). The coking resistance mechanism of the CeAlO_x_/Ni/Ni-foam can be illustrated by the CH_4_ temperature program surface reaction experiment (Fig. 4e), which indicates the decomposition of CH_4_ to H_2_ and carbon on the CeAlO_x_/Ni/Ni-foam is about 50 °C higher than the conventional Ni/CeAlO_x_ catalyst. Moreover, the size of the scraped CeAlO_x_/Ni inverse species before and after cycling experiments maintains a fine dispersion without agglomeration (4.5 nm to 4.9 nm) (Fig. 4f). On the contrary, the Ni NPs over Ni/CeAlO_x_ sinters from 3.2 nm to 10.3 nm after four heating-cooling cycle, which explains the reason for the deactivation of conventional Ni/oxide catalysts.

**Fig. 5:**
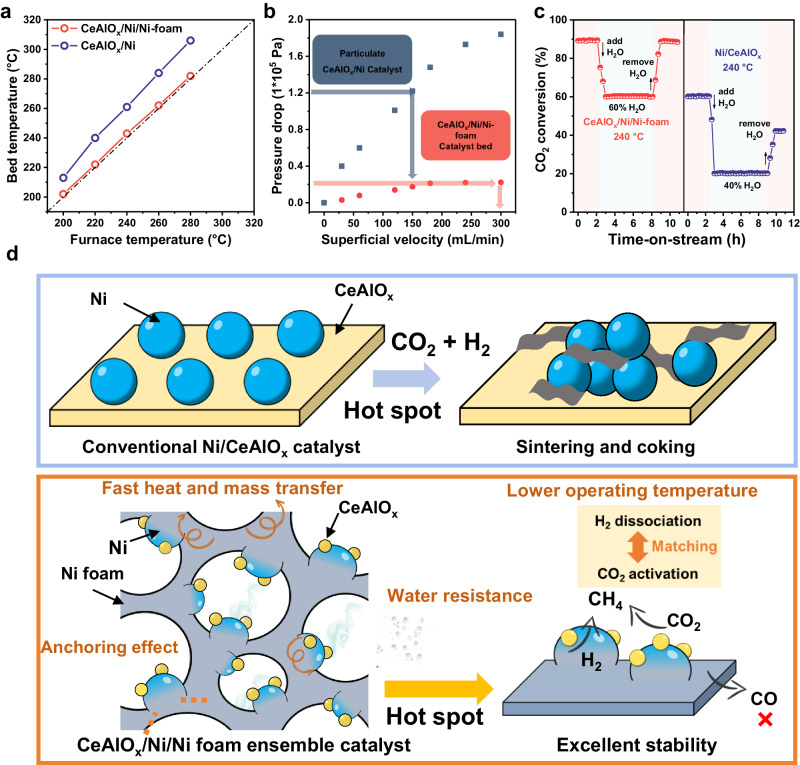
Investigation of the thermal stability of structural catalyst. **a** The comparison of temperature-rising for the Ni-foam-structured CeAlO_x_/Ni catalyst and CeAlO_x_/Ni catalyst; **b** pressure drop against N_2_ gas superficial velocity, CeAlO_x_/Ni/Ni-foam (100 PPI), CeAlO_x_/Ni (60–80 meshes); **c** water resistance test of CeAlO_x_/Ni/Ni-foam catalyst (reaction conditions: 240 °C, GHSV = 10,000 h^−1^, CO_2_:H_2_:N_2_ = 18:72:10, *P* = 0.1 MPa); **d** schematic representation of a Ni-foam skeleton constrained stabilized inverse nickel catalyst and a reference sample.

The excellent thermal stability of structural catalysts compared with the conventional supported catalyst is also probably due to the improved heat and mass transport efficiency. The temperature rise of the catalyst bed is limited below 3 °C in a wide range of reaction temperature and CO_2_ conversion on the CeAlO_x_/Ni/Ni-foam, in contrast, without the Ni foam support, the temperature rise of CeAlO_x_/Ni powder catalyst bed is above 20 °C (Fig. 5a), indicating the diminish of the localized hotspots can be highly due to the construction of structured catalysts. The pressure drops comparison of the CeAlO_x_/Ni /Ni-foam structure catalyst and CeAlO_x_/Ni powder catalyst suggest that the pressure drop of the structured catalyst is only 1/9 of the powder catalysts (0.2 × 10^5 ^Pa at the superficial velocity of 300 mL/min, Fig. 5b). This enhancaced mass transfer efficiency probably also contributes to the hotspot elimination of the nickel foam-based catalyst. Additionally, since steam is one of the main products during methanation reaction, an additional amount of steam is introduced (60 vol% H_2_O) at 240 °C to investigate the water resistent property (Fig. 5c). Ni-structured catalyst loses ~1/3 of its under the reaction condition of 60 vol% H_2_O, but the catalytic activity can be totally recovered after the removal of steam^49,50^. In contrast, the activity of powder catalyst is lost more than 2/3, and only 70% catalytic activity can be recovered after removing steam. The much better water resistence of CeAlO_x_/Ni/Ni-foam structure catalyst can also be attributed to the porous structure that accelerates the diffusion of steam in the reaction.

Based on the performance and cylic stability tests for CO_2_ hydrogenation to methane, the structured catalyst with Ni foam skeleton and well-designed inverse CeAlO_x_/Ni species as active sites is demonstrated to display superior activity, stability and strong adaptability to unsteady operation condition and condensation compared with conventional oxide supported Ni-based catalysts (Fig. 5d). The high thermal conductivity of metal framework and the rich diffusion channels in the structured support successfully eliminate the local hotspots and prevent the accumulation of water surrounding the active sites, which benefits the thermal stability, coke elimination and water resistance. The inverse species which reduces the CO_2_ coverage and accelerates the reduction of CO_2_ and intermediates, not only enhances the activity but also reduces the coke formation due to the successful suppress of CH_4_ decomposition side reactions. Meanwhile, the finely dispersed metal oxide species on inverse MO_x_/Ni composites also enhances the anti-sintering ability of CeAlO_x_/Ni/Ni-foam catalyst and enhances the structure robustness of active species.

**Fig. 6:**
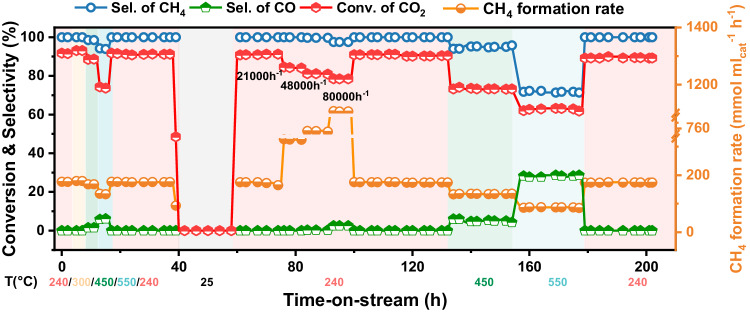
Resistance to fluctuating conditions of CeAlO_x_/Ni/Ni-foam catalysts. Long-term stability test on CeAlO_x_/Ni/Ni foam catalyst. Reaction conditions: 240–550 °C, GHSV = 10,000 h^−1^, CO_2_:H_2_:N_2_ = 18:72:10, *P* = 0.1 MPa.

As CO_2_ methanation is a potential reaction to integrate with unstable and discontinuous hydrogen production from renewable energy, the catalyst developed for the process need to be adaptive to unsteady operation condition and potential steam condensation^51^. Therefore, an unsteady operation condition with waving temperature and space velocity is set to simulate application scenarios and evaluate the stability of CeAlO_x_/Ni/Ni-foam structured catalyst (Fig. 6). No sign of deactivation of catalyst is observed after 200 h time on stream, suggesting the application perspective of CeAlO_x_/Ni/Ni-foam structured catalyst in hydrogen to gas processes.

## Discussion

In summary, a highly active, selective and thermally stable structured catalyst with inverse CeAlO_x_/Ni ensemble active sites loaded on Ni-foam is successfully prepared and applied for the CO_2_ hydrogenation to methane reaction. We demonstrate that the formation of CeAlO_x_ mixed oxide on Ni enhances the oxygen vacancies for CO_2_ activation and simultaneously modulates the surface coverage of CO_2_ and hydrogen, which not only promotes the methanation activity by 14 times but also suppresses the decomposition of CH_4_. Powered with the remarkable heat and mass transport efficiency of 3D Ni-foam and the excellent anchoring effect of Ni(OH)_2_ overlayer prepared by the urea-etching method, the local hotspots are eliminated, and the structure of inverse ensemble is demonstrated to be intact after long-term unsteady operation or treated with steam-rich atmosphere, which overcomes the inherent stability challenges existed in the conventional supported-based catalysts. The development of the CeAlO_x_/Ni/Ni-foam structured catalyst provides rational strategy to construct highly stable and affordable practical catalysts for CO_2_ methanation reaction.

## Methods

### Materials

Analytical grade chemicals including the sodium carbonate (Na_2_CO_3_, 99 wt% purity), sodium hydroxide (NaOH, 99 wt% purity), nickelous nitrate hexahydrate (Ni(NO_3_)_2_·6H_2_O, 98 wt% purity), cerium nitrate hexahydrate (Ce(NO_3_)_2_·6H_2_O, 99 wt% purity) and aluminum nitrate nonahydrate (Al(NO_3_)_3_·9H_2_O, 99 wt% purity) was purchased from Sinopharm Chemical Reagent Co., Ltd. The Ni-foam felt was purchased from Suzhou Taili Material Co. All chemicals were used as received without any further purification.

### Catalyst synthesis

#### Preparation of CeAlO_x_/Ni/Ni-foam catalyst

The Ni(OH)_2_/Ni foam substrate is prepared first. In a typical synthesis procedure, circular Ni foam thin slices (1 g, diameter 6 mm, thickness 1.0 mm, porosity 110 PPI) are cut from Ni foam plates and sonicated in acetone for 20 min to remove surface residual organic impurities. These circular slices are then immersed in a 0.1 M HCl solution at room temperature for an additional 20 min of sonication to remove the surface nickel oxide from the Ni foam, followed by thorough rinsing with deionized water. The cleaned Ni foam thin slices (0.4 g) are transferred to a stainless-steel autoclave lined with a 50 mL polytetrafluoroethylene (PTFE) container, which contains a 35 mL solution of urea (6.3 mmol). After hydrothermal treatment at 160 °C for 8 h, the Ni foam coated with deep green Ni(OH)_2_ crystals is rinsed with deionized water and dried under vacuum at 80 °C for 12 h. A solution containing Al(NO_3_)_3_·9H_2_O (0.875 mmol), Ce(NO_3_)_2_·6H_2_O(0.218 mmol), and urea (6.5 mmol) is prepared (35 mL), and then the obtained solution is stirred for about 60 min. Subsequently, the resulting solution and Ni(OH)_2_/Ni foam thin slices (0.4 g) are transferred to a Teflon-lined autoclave reactor (100 mL), subjected to hydrothermal treatment at 180 °C for 12 h. After cooling to room temperature, the sample is washed with ethanol and deionized water, dried under vacuum at 60 °C for 12 h, and finally calcined at 400 °C for 3 h to obtain the CeAlO_x_/NiO/Ni foam catalyst.

#### Preparation of Ni/CeAlO_x_ catalyst

The Ni/CeAlO_x_ catalyst is prepared by a coprecipitation method. Briefly, a solution containing Al(NO_3_)_3_·9H_2_O (5 mmol), Ce(NO_3_)_2_·6H_2_O (1.25 mmol), and Ni(NO_3_)_2_·6H_2_O (2.68 mmol) is prepared (100 mL), and then the aqueous metal precursor solutions are added dropwise to a precipitating solution of Na_2_CO_3_ and NaOH at vigorous stirring conditions. The resulting solution is stirred for 1 h, then maintain the pH to 10 by adding 3 M NaOH solution. After that, the precipitated mixture is aged at 65 °C in the reactor for 18 h to promote the crystallization of metals. Finally, the solid precipitate is filtered out be washed with ultrapure water many times to reduce the pH of the mixture to neutral. The obtained solid is dried at 110 °C overnight, and further calcinated at 400 °C in air^52^.

### Catalytic evaluation

The performance evaluation of CO_2_ hydrogenation to methane is performed in an atmospheric fixed-bed reactor. The prepared catalyst sheets (0.15 g, diameter 6 mm) are loaded into a quartz tube (inner diameter = 6 mm and length = 60 cm) and put into the reactor. The catalyst is preprocessed in 20% H_2_ at 450 °C for 3 h, cooled to the reaction temperature (160–300 °C), then the reaction gas (CO_2_:H_2_:N_2_ = 18:72:10) is fed into the reactor. The actual temperature of the catalyst bed is measured using a thermocouple located at the middle of the catalyst bed. Gas-phase products are analyzed using a gas chromatograph (GC-8860, Agilent) equipped with a thermal conductivity detector, Porapak Q and 5 A molecular sieve columns. The definitions of CO_2_ conversion, CH_4_ selectivity, carbon balance, and CH_4_ STY are given by the following equations:

1 $$X\left({{CO}}_{2}\right) \%=\frac{F * {C}_{{in}}\left({{CO}}_{2}\right)-{F * C}_{{out}}\left({{CO}}_{2}\right) * \frac{{A}_{{in}\left({N}_{2}\right)}}{{A}_{{out}\left({N}_{2}\right)}}}{F * {C}_{{in}}({{CO}}_{2})}$$

2 $$S({{CH}}_{4}) \%= \frac{n({{CH}}_{4})}{\sum n({products})}$$

3 $$S({CO}) \%= \frac{n({CO})}{\sum n({products})}$$

4 $${STY}({{CH}}_{4})({{mmol}}_{{{CH}}_{4}}\cdot {{ml}}_{{cat}}^{-1}\cdot {h}^{-1})=\frac{{n}_{{in}}\left({{CO}}_{2}\right)\cdot X({{CO}}_{2})\cdot S\left({{CH}}_{4}\right)\cdot 16\cdot 60}{22.4\cdot {V}_{{cat}}}$$

where *F* denotes the gas flow into the reactor, C denotes the concentration, *A* denotes the gas chromatographic peak area, *V*_cat_ denotes the volume of catalyst and *n* denotes the amount of substance.

The Arrhenius plots were created at a high GHSV of 15,000–40,000 h^−1^ to ensure that the concentration of carbon dioxide produced remained below 15%. This was achieved due to the insignificant influence of heat and mass transfer in this region. Additionally, differential mass-normalized reaction rates were calculated in the kinetic regime.

### Catalyst characterization

#### Inductively coupled plasma-optical emission spectrometer

The ICP-OES results are performed on Varian ICP-OES 720. Sample preparation: A certain number of samples are weighed into a PTFE container, added with 5 mL concentrated nitric acid, 3 mL HCl, 1 mL HF and 2 mL H_2_O_2_, sealed in a microwave digestion furnace, heated at 1200 W for 20 min to 130 °C, kept for 5 min, heated for 20 min to 180 °C, kept for 40 min, and cooled to room temperature. Test: The cooled solution is transferred to a 25 mL plastic volumetric bottle, and filled with deionized water. The dissolved solution is tested sequentially, and the diluted solution beyond the curve is tested again. Standard test solution: the standard solution is a national standard material, and the curve concentration points are 0, 0.5, 1.0, 2.0, 5.0 mg/L, respectively.

#### X-ray diffraction

XRD is used to determine the phase composition and estimate the particle size of the catalyst. The testing is conducted using a Cu-Kα excitation source with a scanning range of 2θ = 10° ~ 80°, a scanning speed of 20°/min, and a step size of 0.0167. The phase analysis is conducted by referring to the standard powder diffraction cards. The particle size of Ni is calculated using the Scherrer equation.

#### Surface area measurement

N_2_ physical adsorption testing is conducted on the BSD-PS2 instrument. Prior to the testing, the sample is subjected to a vacuum degassing at 200 °C for 4 h, followed by N_2_ adsorption-desorption testing under liquid nitrogen cooling (−196 °C) conditions. The determination of the specific surface area and distribution of pore sizes is accomplished through utilization of the Brunauer-Emmett-Teller (BET) method for calculation, in conjunction with analysis of the desorption curve using the Barrett-Joyner-Halenda (BJH) technique.

#### H_2_ temperature-programmed reduction (H_2_-TPR)

H_2_-TPR is conducted on the BELCAT-B instrument. A sample of 50 mg is weighed and pretreated in a flowing pure He gas (30 mL/min) for 1 h at 130 °C. After the sample is cooled to room temperature., a flow of H_2_/Ar (10/90) gas (30 mL/min) is introduced. The temperature is then ramped from 50 °C to 700 °C with a heating rate of 10 °C/min for the temperature-programmed reduction process. The consumption of hydrogen is recorded by a thermal conductivity detector.

#### CO_2_ temperature-programmed desorption (CO_2_-TPD)

CO_2_-TPD is conducted on the Microtrac BEL Cat II instrument. The catalyst (50 mg) is pretreated at 450 °C for 180 min in 20% H_2_ (heating rate of 5 °C/min), followed by cooling to 50 °C and purge with He for 30 min. Then, the catalyst is treated in CO_2_/He (10/90) for 60 min, followed by a 40-min purge with He to remove unabsorbed and physically adsorbed CO_2_. After the baseline has been stabilized, the temperature is gradually increased from room temperature to 800 °C at a heating rate of 10 °C/min in order to facilitate the desorption of CO_2_.

#### Temperature-programmed oxidation (TPO) of spent catalysts

The catalysts, after stability test are exposed to 20% O_2_/Ar (50 mL/min) at ambient temperature purge for 30 min, the fixed-bed reactor is heated to 700 °C with a rate of 10 °C/min and then held for 10 min. The CO and CO_2_ are quantified by mass spectrum analyzer (DECRA), but CO_2_ is the major product^53^.

#### Scanning electronic microscopy

The samples were analyzed using a high-resolution field emission scanning electron microscope (FE-SEM, HITACHI Regulus 8100) operating at an acceleration voltage of 20 kV. Following that, the distribution of elements was determined utilizing EDX (Oxford Ultim Max 65).

#### Transmission electron microscope

TEM is conducted using a FEG-TEM instrument (Tecnai G2 F30 S-Twin) operating at 300 kV. The samples are sparsely dispersed in ethanol and subsequently deposited onto copper grids coated with amorphous carbon films, followed by desiccation for TEM observations^54^.

#### Scanning transmission electron microscope

The Thermo Scientific Spectra 300 Double-Corrected Transmission Electron Microscope, equipped with a Gatan Imaging Filter, was utilized to conduct the STEM and EDX experiments. The point of scanning for elemental mapping within STEM-EDX was determined at 150×150. The predetermined operating parameters necessitated the application of an acceleration voltage of 300 kV. To facilitate analysis and evaluation of the findings, the surface active phase CeAlOx/Ni from the reduced passivated nickel foam catalyst was extracted prior to TEM sample preparation for characterization.

#### X-ray photoelectron spectroscopy

X-ray Photoelectron Spectroscopy analysis is performed on a ThermoFischer ESCALAB 250Xi equipped with an in situ reactor. The specific parameters are as follows: excitation source using Al Kalpha radiation (*hv* = 1486.6 eV); analysis chamber vacuum level of 8 × 10^−10^ mbar; working voltage of 12.5 kV; filament current of 16 mA; and signal accumulation for ~10 cycles. The Passing Energy is set to 30 eV with a step size of 0.1 eV. The specific operational procedure is as follows: the catalyst sample, in the form of a disc, is placed inside the reactor chamber. It is pretreated for 1 h at a set temperature in an H_2_/N_2_ atmosphere (20 vol% H_2_) with a flow rate of 20 mL/min. After cooling to room temperature, the sample is transferred to the measurement chamber without exposure to air. The measurement chamber is evacuated to a vacuum level below 8 × 10^−10^ mbar before conducting the analysis. Charging correction of the binding energy is performed using C1*s* (284.6 eV) as a reference.

#### Raman spectroscopy analysis

Raman spectra are obtained using the Renishaw In Via Reflex spectrometer with a 532 nm laser excitation source. The scanning range is set from 200 to 1800 cm^−1^ with an accuracy of 2 cm^−1^. The scan test is considered complete when consistent results are obtained from at least three positions on each sample.

#### Oxygen pulse titration (O_2_-PT)

For the O_2_ pulse experiments of NiO/Ni-foam, Al_2_O_3_/NiO/Ni-foam and CeAlO_x_/NiO/Ni-foam catalysts are pretreated at 450 °C for 3 h under H_2_ flow (20 vol% H_2_/N_2_, 40 mL/min), purged 10 min with He and heated to 500 °C. Then the 1% O_2_ pulse experiments are repeated until the TCD peak intensity is equal.

6 $${O}_{{vacancy}}=\frac{V\left({O}_{2}\right)\cdot {SF}/22400}{{\omega }_{{oxide}}\cdot {m}_{{cat}}}$$

where SF represents the stoichiometry factor, V(O_2_) is the consumption of O_2_ (deduct the Ni/Ni-foam consumption), *ω*_oxide_ is the oxide mass fraction (%), and *m*_cat_ is the mass of the catalyst (g).

#### Temperature-programmed surface reaction-mass spectrum

The test procedure for CH_4_ dissociation: 100 mg of sample, pretreat it at 450 °C for 3 h under 40 mL∙min^−1^ 20% H_2_/Ar purge. Then cool down to room temperature (approximately 25 °C), and switch the 20% H_2_/Ar to 25 mL∙min^−1^ 10% CH_4_/Ar to record mass baseline. After the baseline is stable, the temperature is increased to 750 °C with a heating rate of 10 °C∙min^−1^, while the mass spectrum is recorded at the same time^55^.

#### In situ diffuse reflectance infrared flourier transform spectroscopy

In situ DRIFTs measurements are performed by using an FTIR spectrometer (Bruker Vertex 80) equipped with a Harrick cell and a liquid nitrogen-cooled MCT detector, along with an RGA detector for the outlet gas analysis. The CeAlO_x_/Ni/Ni-foam and Al_2_O_3_/Ni/Ni-foam catalysts are reduced in 10 mL min^−1^ (H_2_/Ar = 20/80) gas flow at 450 °C for 3 h, and then cooled down to 180 °C and purged with Ar for 30 min. The temperature of in situ DRIFTs is chosen to be 180 °C instead of 220 °C, in order to better observe the intermediate species at low activity. 1 min is averaged for each spectrum, which is recorded at a resolution of 4 cm^−1^. Prior to each experiment, background is collected at Ar and 180 °C. Subsequently, the gas flow is changed to 80% H_2_/20% CO_2_ (10 mL min^−1^, 0.1 MPa) at the same temperature, and the spectra are collected simultaneously. The transmittance is obtained by dividing the collected sample reflectance spectrum by the background spectrum, then spectrum is converted to Kubelka–Monk. After 90 min reaction in an 80% H_2_/20% CO_2_ atmosphere, the inlet is switched to 80% H_2_/20% Ar (10 mL min^−1^) at the same temperature. At the same time, DRIFTs spectra are recorded to monitor the change of intensity of different surface species for another 90 min.

### Supplementary information

Supplementary Information

Peer Review File

### Source data

Source Data

## Data Availability

The data that support the plots within this paper and another finding of this study are available from the corresponding author upon reasonable request. Source data are provided as a Source Data file. Source data are provided in this paper.
