# Supplementary material for: Thermally stable Ni foam-supported inverse CeAlOx/Ni ensemble as an active structured catalyst for CO2 hydrogenation to methane
Source: Nat Commun. 2024 Apr 10;15:3115. doi: 10.1038/s41467-024-47403-4 (PMC11006838; doi:10.1038/s41467-024-47403-4)
Supplement: Supplementary file 1 — Supplementary Information [file 41467_2024_47403_MOESM1_ESM.pdf]

Supplementary Information

Thermally Stable Ni Foam-Supported Inverse $CeAlO_x$/Ni Ensemble as an Active Structured Catalyst for CO_2 hydrogenation to Methane

Xin Tang[1,2+], Chuqiao Song[1,2+], Haibo Li[1,2], Wenyu Liu[1,2], Xinyu Hu[1], Qiaoli Chen[1], Hanfeng Lu[1], Siyu Yao[3], Xiao-nian Li[1,2], Lili Lin[1,2]**

[1]Institute of Industrial Catalysis, State Key Laboratory of Green Chemistry Synthesis Technology, College of Chemical Engineering, Zhejiang University of Technology, Hangzhou, Zhejiang 310014, China.

[2]Zhejiang Carbon Neutral Innovation Institute & Zhejiang International Cooperation Base for Science and Technology on Carbon Emission Reduction and Monitoring, Zhejiang University of Technology, Hangzhou 310014, China.

[3]Key Laboratory of Biomass Chemical Engineering of Ministry of Education, College of Chemical and Biological Engineering, Zhejiang University, Hangzhou, 310027 China

[†] These authors contributed equally to this work.

* Email: linll@zjut.edu.cn; yaosiyu@zju.edu.cn

Supplementary Figures and Tables

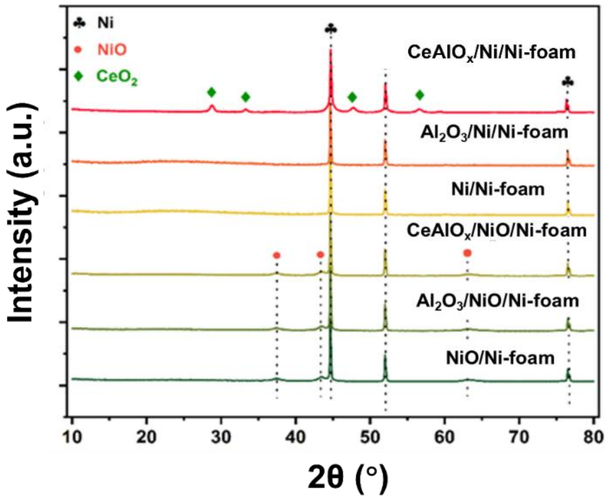

Supplementary Fig 1. XRD patterns of the $CeAlO_x$/NiO/Ni-foam, $CeAlO_x$/Ni/Ni-foam, Al_2O_3/NiO/Ni-foam, Al_2O_3/Ni/Ni-foam, NiO/Ni-foam and Ni/Ni-foam samples.

Supplementary Note 1: The characteristic peaks of NiO appear in the calcined samples of $CeAlO_x$/NiO/Ni-foam, Al_2O_3/NiO/Ni-foam, and NiO/Ni-foam, which will disappear after reduction, confirming that the etched NiO layer could be reduced to metallic Ni. The characteristic peak of CeO_2 could be observed after reduction, confirming the increased crystallinity of oxide. The characteristic peaks of Al_2O_3 could not be observed on $CeAlO_x$/NiO/Ni-foam, $CeAlO_x$/Ni/Ni foam, Al_2O_3/NiO/Ni-foam and Al_2O_3/Ni/Ni-foam samples, confirming that Al_2O_3 is primarily amorphous.

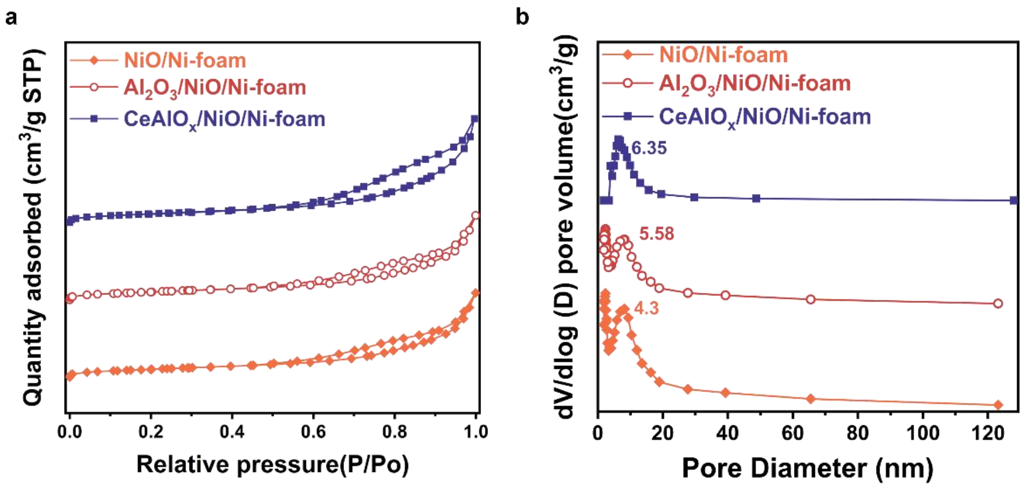

Supplementary Fig 2. N_2 physical adsorption testing. a. N_2 adsorption-desorption isotherms; b. pore size distributions of the MO_x/NiO/Ni-foam samples.

Supplementary Table 1. Physicochemical characteristics of the MO_x/NiO/Ni foam samples.

Samples	NiO Loading[a] (wt%)	Al Loading[b] (wt%)	Ce Loading[b] (wt%)	S_{BET}[c] (m^2/g)	Total volume[c] (cm^3/g)	Average pore diameter[c] (nm)
NiO/Ni-foam	20	0	0	13.7	0.083	4.3
Al_2O_3/NiO/Ni-foam	19.2	4.1	0	43.2	0.178	5.5
$CeAlO_x$/NiO/Ni-foam	20.3	2.5	2.4	55.4	0.219	6.2
$CeAlO_x$/Ni/Ni-foam-used	--	2.6	2.3	35.2	0.202	5.2
Ni/$CeAlO_x$	13	24	31	130	0.35	8.5

[a] Estimated by H_2-TPR according to the reaction: $H_2 + NiO = Ni + H_2O$. NiO: The surface of nickel foam is etched by urea to form $Ni(OH)_2$ layer, which is calcined to form NiO

$$\boldsymbol{NiO\ wt\%} = \frac{\boldsymbol{n_{H2} * M_{NiO}}}{\boldsymbol{m_{catalyst}}} \qquad (\mathbf{7})$$

[b] Determined by ICP-OES.

$$\boldsymbol{n\ wt\%} = \frac{\boldsymbol{m_n}}{\boldsymbol{m_{catalyst}}} \qquad (\mathbf{8})$$

where n_{H2} denotes the hydrogen consumption, m_n denotes the quality of Al and Ce obtained by ICP-OES test, $m_{catalyst}$ denotes the total amount of MOx/Ni(O)/Ni-foam structure.

[c] Determined by BET method.

Footnote: The wt% calculation of NiO, Al and Ce is based on the total amount of MO_x/Ni(O)/Ni-foam structure.

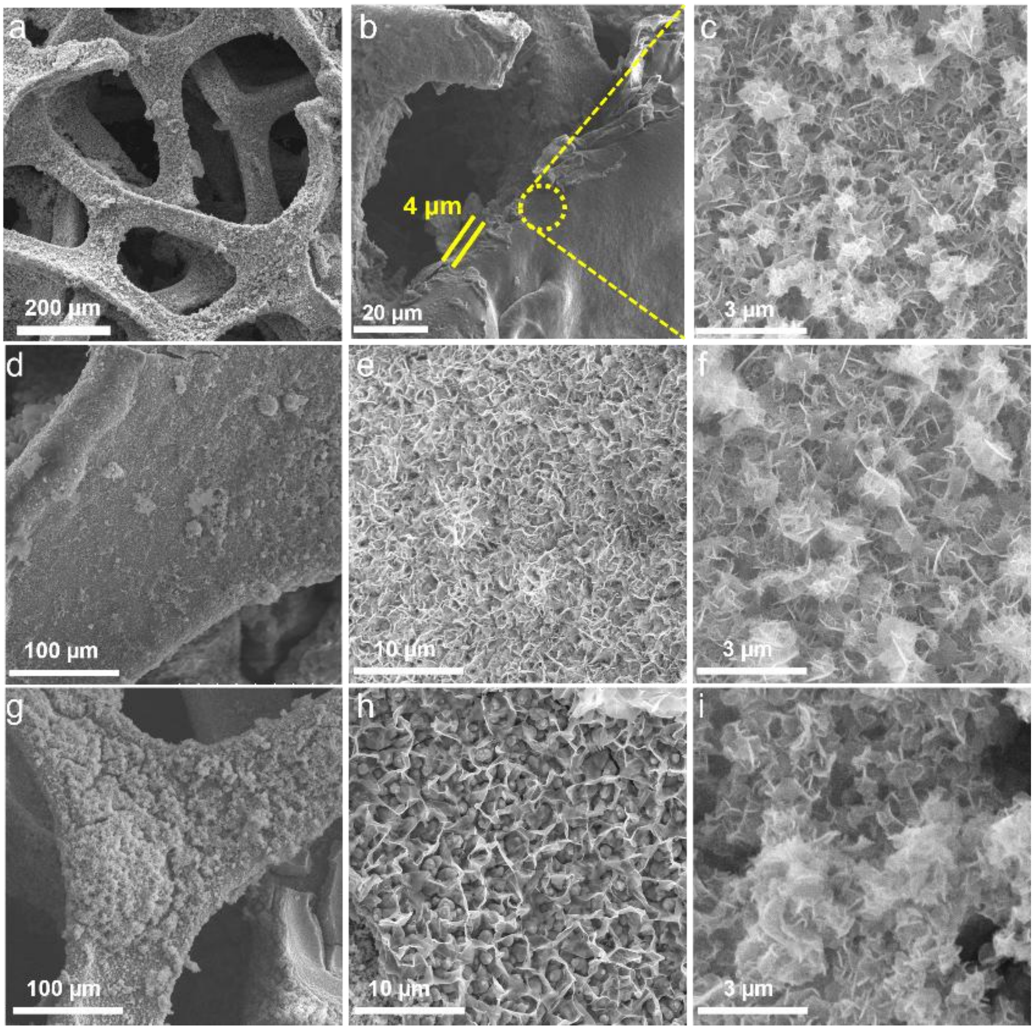

Supplementary Fig 3. SEM characterization image of structured catalysts. a-c. SEM images of NiO/Ni-foam; d-f. SEM images of Al_2O_3/NiO/Ni-foam; g-i. SEM images of $CeAlO_x$/NiO/Ni-foam catalysts.

Supplementary Note 2: The $Ni(OH)_2$ nanosheets, cultivated through the metal-induced chemical etching process, exhibit a substantial surface area and abundant defect sites on the nickel foam surface, thereby obviating the time-consuming physical deposition process. Furthermore, owing to its oxygen-containing functional groups, $Ni(OH)_2$ plays a pivotal role in anchoring and facilitating the growth of nano-oxides. SEM images reveal that after calcination, tightly self-assembled NiO nanosheets form an inverse interface with hydrothermally synthesized nano-oxides on the nickel foam surface.

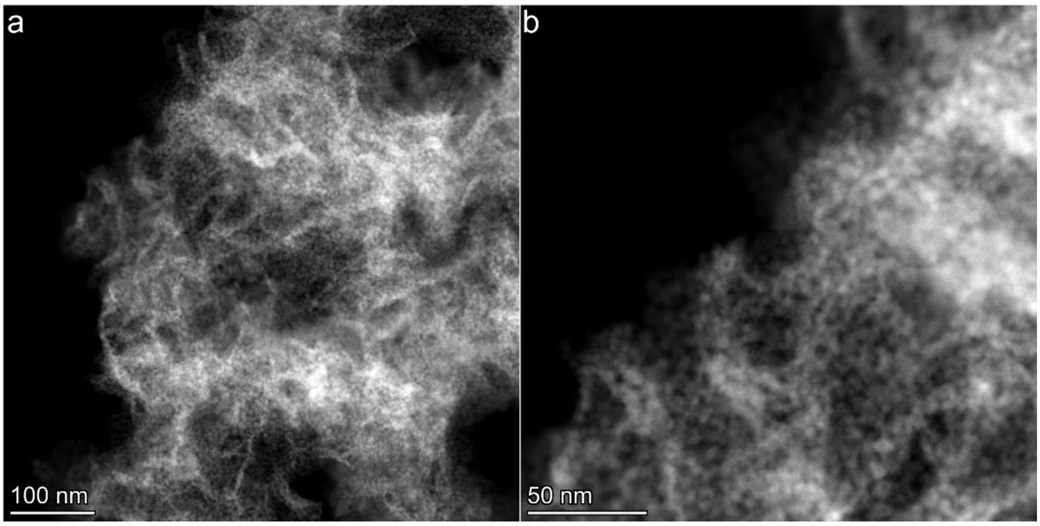

Supplementary Fig 4. Representative HAADF-STEM images of $CeAlO_x$/Ni/Ni-foam after reduction. a Low-magnification TEM image for $CeAlO_x$/Ni/Ni-foam catalyst; b enlarged TEM image for $CeAlO_x$/Ni/Ni-foam catalyst.

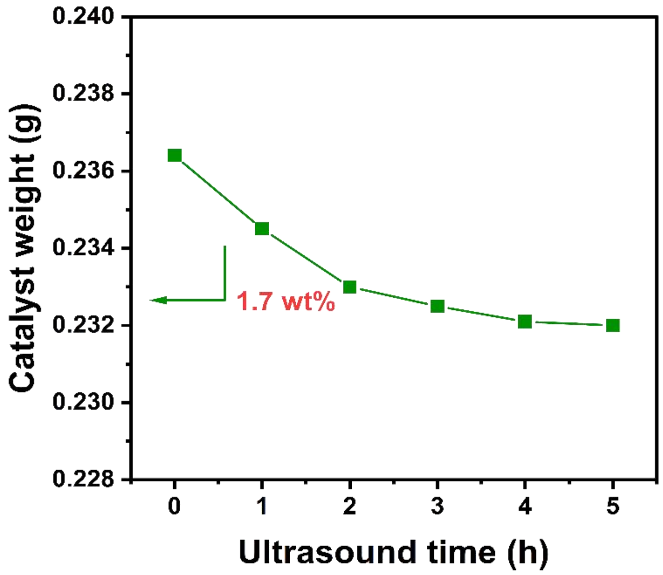

Supplementary Fig 5. Weight loss of the $CeAlO_x$/NiO/Ni-foam catalyst after an ultrasonic treatment in methanol for 4 hours.

Supplementary Note 3: Following 4 hours of ultrasonic treatment, the active layer demonstrates an impressive retention rate exceeding 98%, indicating robust adhesion.

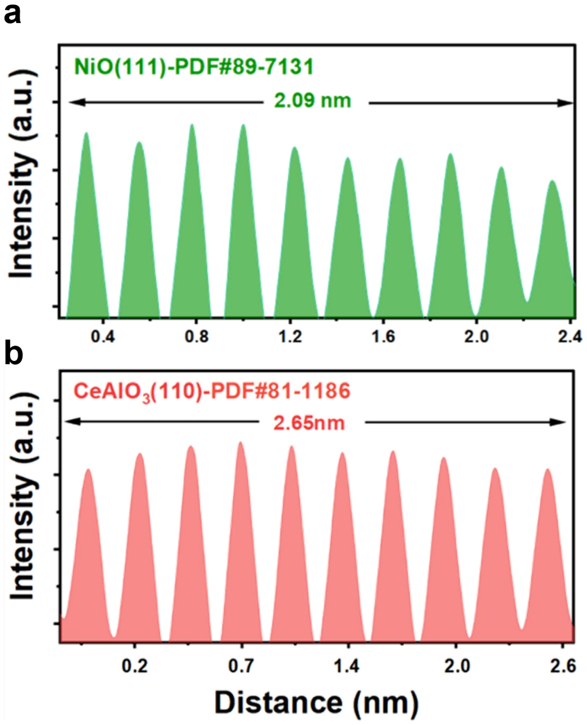

Supplementary Fig 6. The analysis of lattice fringes of the HAADF-STEM images. a. Intensity profiles measured from NiO(111) of Fig. 1f; b. intensity profiles measured from $CeAlO_3$(110) of Fig. 1f.

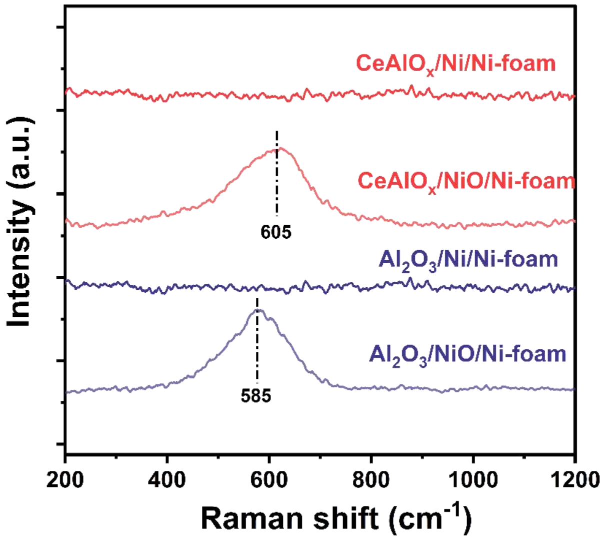

Supplementary Fig 7. Raman spectra of the $CeAlO_x$/NiO/Ni-foam, $CeAlO_x$/Ni/Ni-foam, Al_2O_3/NiO/Ni-foam and Al_2O_3/Ni/Ni-foam samples.

Supplementary Note 4: The vibration peaks around ~585 cm^{-1} and ~605 cm^{-1} which corresponds to Al-O-Ni and Ce-O-Ni are observed in the Al_2O_3/NiO/Ni-foam and $CeAlO_x$/NiO/Ni-foam samples before reduced. Reduction process leads to the disappearance of above-mentioned peaks, indicating that NiO on the surface is fully reduced to metallic Ni, which is consistent with the XRD results (Fig. S1).

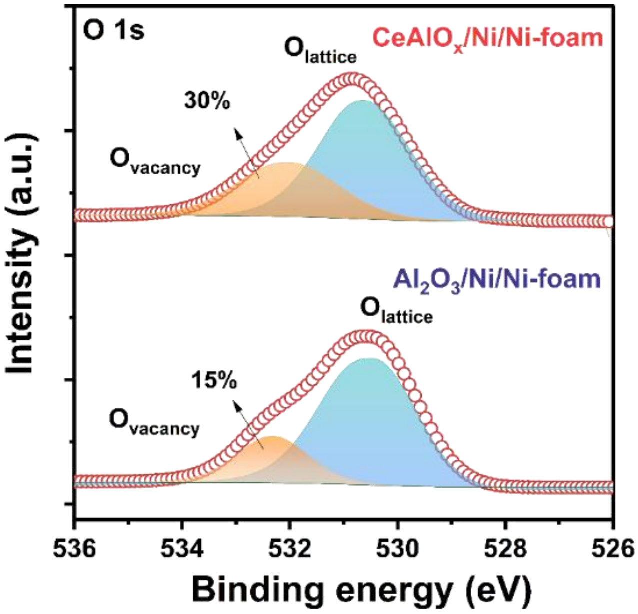

Supplementary Fig 8. In situ XPS spectra of O 1s of $CeAlO_x$/Ni/Ni-foam and Al_2O_3/Ni/Ni-foam catalysts.

Supplementary Note 5: The detailed O1s spectra are deconvoluted into two peaks, namely lattice oxygen species ($O_{lattice}$: O^{2-}) centered at 530.1−530.4 eV, and the surface oxygen species ($O_{surface}$: O^{2-}, O_2^{2-}, or O^-) centered at 531.3−531.5 eV.[1, 2] The reduction of oxide could generate abundant oxygen vacancies that could be reflected by the ratio of $O_{surface}/(O_{lattice}+O_{surface})$. The ratios of $O_{surface}/(O_{lattice}+O_{surface})$ over $CeAlO_x$/Ni/Ni-foam and Al_2O_3/Ni/Ni-foam catalyst are ~30% and ~15%, respectively, suggesting a high content of surface oxygen vacancies of $CeAlO_x$/Ni/Ni-foam catalyst.

Supplementary Table 2. The value of binding energy for the XPS fitting result.

Sample	Element			Binding Energy (eV)
$CeAlO_x$/Ni/Ni-foam	Ce	Ce^{3+}	3d	881.18、885.1、899.4、903.6、
		Ce^{4+}	3d	882.6、898.1、888.6、907.2<br>901.4、916.6
	O	$O_{lattice}$	1s	530.2
		$O_{surface}$	1s	531.2
Al_2O_3/Ni/Ni-foam	O	$O_{lattice}$	1s	530.1
		$O_{surface}$	1s	531.4

Supplementary Table 3. CO_2 adsorption capacity of Ni-based catalysts.

Catalysts	CO_2 desorption (μmol_{CO2} mL_{foam}^{-1})			
	Weak (<150 °C)	Medium (150-450 °C)	Strong (>450 °C)	Total
Ni/Ni-foam	0	5	40.2	45.2
Al_2O_3/Ni/Ni-foam	4.2	97.8	6.2	108.2
$CeAlO_x$/Ni/Ni-foam	8.9	141.1	1.5	151.5
Ni/$CeAlO_x$	8	62.0	304.2	374.2
Ni/Al_2O_3	1.5	13.5	302	317.0

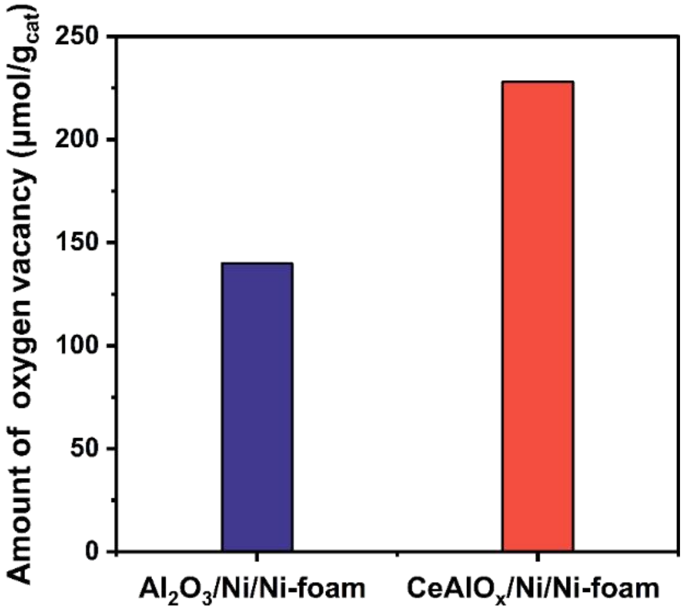

Supplementary Fig 9. The amount of oxygen vacancy of $CeAlO_x$/Ni/Ni-foam and Al_2O_3/Ni/Ni-foam catalysts.

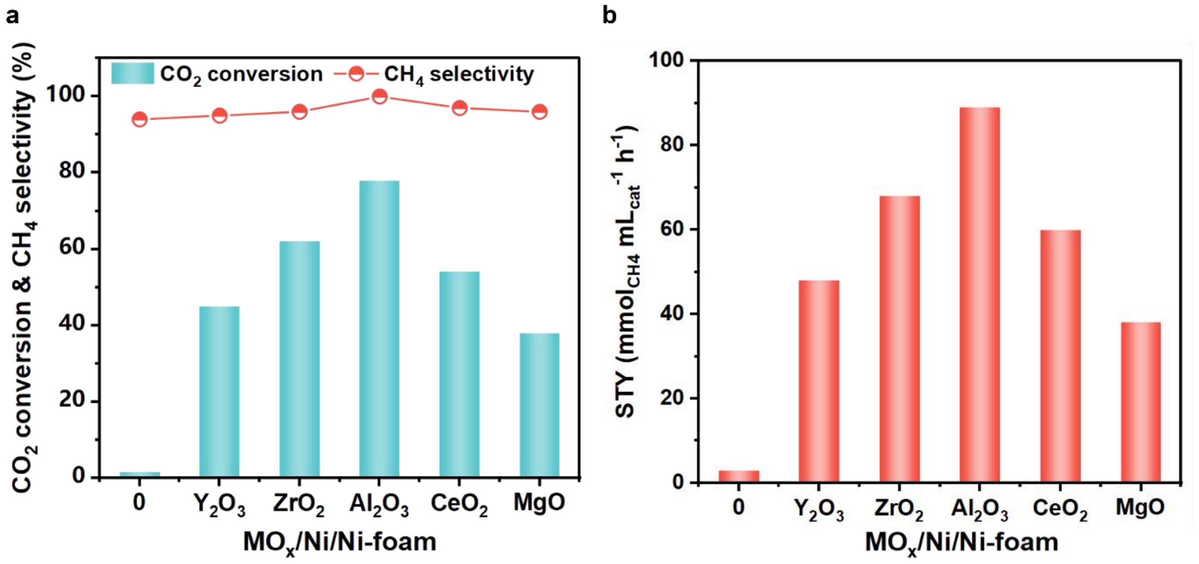

Supplementary Fig 10. Comparison of activity of MO_x/Ni/Ni-foam catalysts under given conditions. a. The CO_2 conversion and CH_4 selectivity of MO_x/Ni/Ni-foam catalysts; b. the CH_4 STY of MO_x/Ni/Ni-foam catalysts. Reaction conditions for the catalytic test: GHSV=10,000 h^{-1}, 240 °C, CO_2:H_2:N_2=18:72:10, P = 0.1 MPa.

Supplementary Note 6: Compared to the Ni/Ni-foam catalyst, the catalysts with oxide modification show better performance of methane production. As shown in Fig. S8, the Al_2O_3/Ni/Ni-foam is the most active catalyst with CO_2 conversion rate of ~80% and CH_4 selectivity ~99.9% at 240 °C, which approaches to the thermodynamic equilibrium conversion.

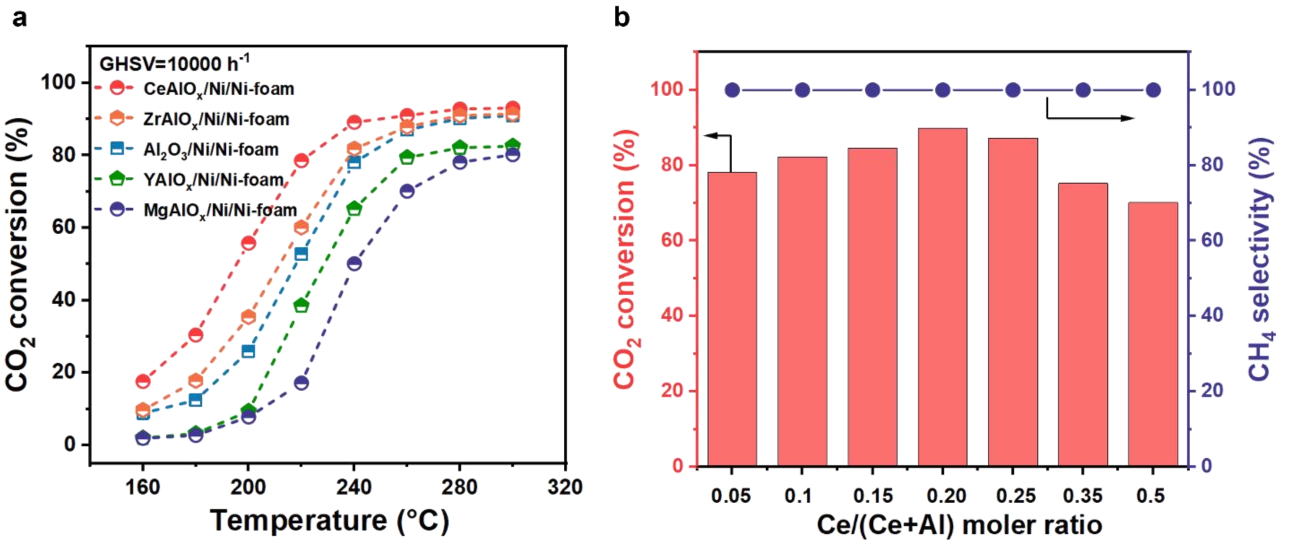

Supplementary Fig 11. Selection and dosage modulation of catalyst promoters. a. Temperature-dependent activities of Al_2O_3/Ni/Ni-foam catalysts with different promoters; b. temperature-dependent activities of $CeAlO_x$/Ni/Ni-foam catalysts with different Ce/(Ce+Al) ratios. Reaction conditions for the catalytic test: GHSV=10,000 h^{-1}, 160-320 °C, CO_2:H_2:N_2=18:72:10, P = 0.1 MPa.

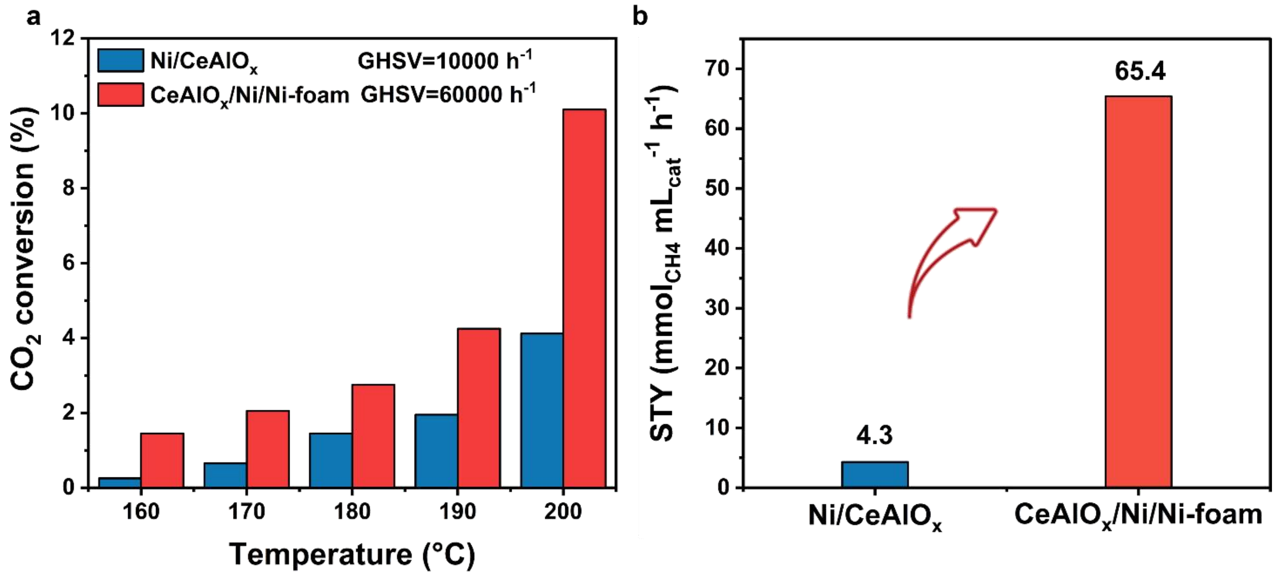

Supplementary Fig 12. Comparison activity results of catalysts in kinetic region. a. Comparison results of CO_2 Conversion of Ni/$CeAlO_x$ and $CeAlO_x$/Ni/Ni-foam catalysts in kinetic region; b. Comparison results of CH_4 STY of Ni/$CeAlO_x$ and $CeAlO_x$/Ni/Ni-foam catalysts in kinetic region. Reaction conditions for the catalytic test to the kinetic range: GHSV=60,000 h^{-1}, 160-200 °C for $CeAlO_x$/Ni/Ni-foam, GHSV=10,000 h^{-1}, 160-200 °C for Ni/$CeAlO_x$ catalyst, P = 0.1 Mpa.

Supplementary Table 4. Comparison of activity of $CeAlO_x$/Ni/Ni-foam and Ni/$CeAlO_x$ catalysts in kinetic region.

Catalyst	Temperature (°C)	GHSV (h^{-1})	X_{CO2} (%)	S_{CH4} (%)	CH_4-STY (mmol/mL_{cat}/h)
$CeAlO_x$/Ni/Ni-foam	200	60000	10.6	100	65.4
Ni/$CeAlO_x$	200	10000	4.2	100	4.3

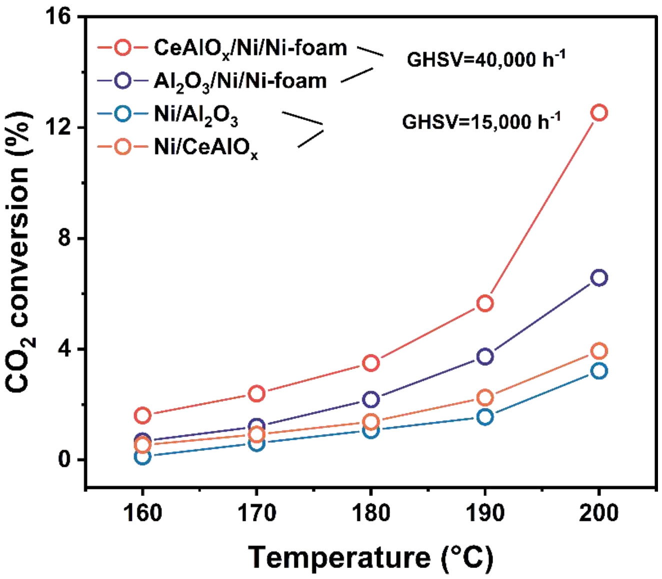

Supplementary Fig 13. Details of the Ea calculation for $CeAlO_x$/Ni/Ni-foam and Ni/$CeAlO_x$ catalysts. The activity of 160-190 °C was used for Ea calculation of $CeAlO_x$/Ni/Ni-foam and AlO_x/Ni/Ni-foam catalyst, while the activity of 170-200 °C was used for Ea calculation of Ni/Al_2O_3 and Ni/$CeAlO_x$ catalysts. Reaction conditions:160-200 °C, CO_2:H_2:N_2=18:72:10, P = 0.1 MPa. In order to control the reaction in kinetic region to calculate the Ea more accurately, the CO_2 conversion is controlled below 10% by regulating GHSV from 15,000 to 40,000 h^{-1}.

Supplementary Table 5. Details of H_2 and CO_2 reaction order test[a].

Order	Catalyst	$CO_2/H_2/N_2$	X_{CO2}/%	CH_4 STY(mmol/mL$_{foam}$/h)
H_2	$CeAlO_x$/Ni/Ni-foam	8/4/88	2.36	3.38
		8/8/84	3.03	4.33
		8/16/76	3.64	5.19
		8/24/68	4.23	6.05
	Al_2O_3/Ni/Ni-foam	8/4/88	1.93	2.76
		8/8/84	2.62	3.74
		8/16/76	3.27	4.66
		8/24/68	3.64	5.21
	Ni/$CeAlO_x$	8/4/88	0.93	1.32
		8/8/84	1.67	2.38
		8/16/76	2.94	4.21
		8/24/68	4.01	5.73
	Ni/Al_2O_3	8/4/88	0.84	1.20
		8/8/84	1.51	2.16
		8/16/76	2.66	3.81
		8/24/68	3.63	5.19
	$CeAlO_x$/N/Ni-foam	4/16/80	5.97	4.26
		8/16/76	3.40	4.85
		12/16/72	2.61	5.58
		16/16/68	2.08	5.93
	Al_2O_3/Ni/Ni-foam	4/16/80	5.35	3.82
		8/16/76	3.20	4.57
		12/16/72	2.43	5.21
		16/16/68	1.97	5.64
	Ni/$CeAlO_x$	4/16/80	5.24	3.74
		8/16/76	2.67	3.82

CO_2		12/16/72	1.85	3.97
		16/16/68	1.42	4.06
	Ni/Al_2O_3	4/16/80	5.19	3.71
		8/16/76	2.62	3.74
		12/16/72	1.80	3.86
		16/16/68	1.38	3.94

a: Reaction conditions for the H_2 and CO_2 reaction order tests: GHSV=40,000 h^{-1}, 180 °C for $CeAlO_x$/Ni/Ni-foam and Al_2O_3/Ni/Ni-foam catalysts, 220 °C for 30 mol% Ni/$CeAlO_x$ and Ni/Al_2O_3 catalyst, P = 0.1 Mpa. The purpose of diluting CO_2 reaction gas is to convert CO_2 in the kinetic region while also eliminating the effect of hot-spot.

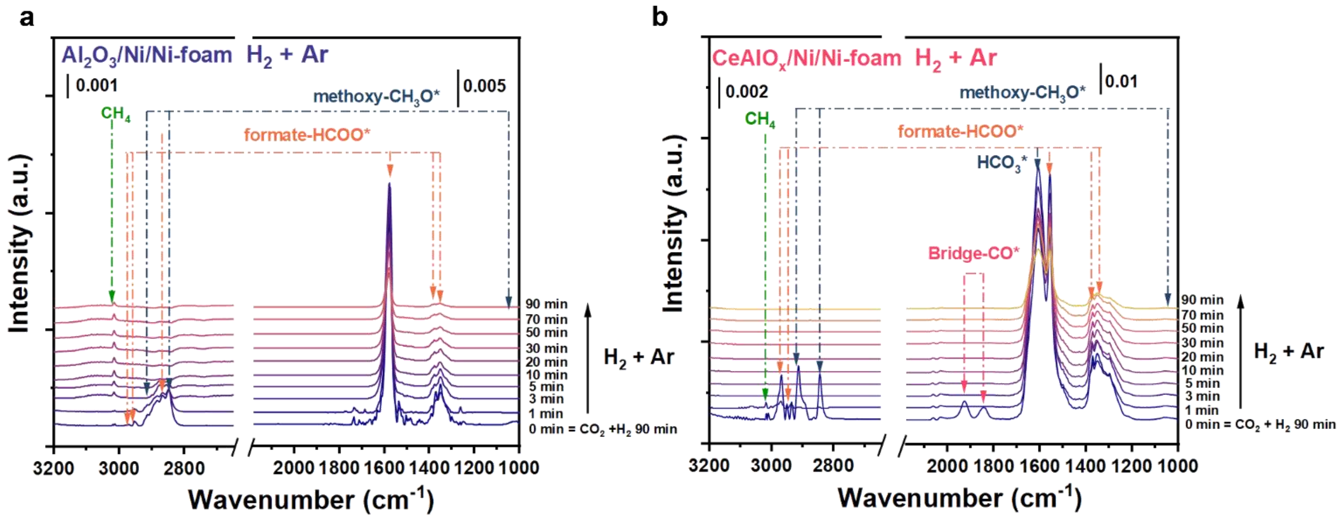

Supplementary Fig 14. Surface intermediates investigation by in situ DRIFTs characterization. In-situ DRIFTs of the H_2 atmosphere on (a) Al_2O_3/Ni/Ni-foam and (b) $CeAlO_x$/Ni/Ni-foam catalysts (reaction condition： pretreat 90 mins in 80% H_2/20% CO_2 atmosphere at 180 °C and the inlet is switched to 80% H_2/20% Ar and maintained at the same temperature for 90 min).

Supplementary Table 6. Comparison of the catalytic hydrogenation activities of $CeAlO_x$/Ni/Ni-foam, Al_2O_3/Ni/Ni-foam and related catalysts.[3-26]

Catalysts	T/°C	GHSV	H_2/CO_2 Inert	X_{CO_2}/%	S_{CH_4}/%	CH_4-STY /mmol·g^{-1}·h^{-1}	Refs.
$CeAlO_x$/Ni/Ni-foam	240	15,000	4	90	100	106.3	This work
$CeAlO_x$/Ni/Ni-foam	240	180,000	4	80	98	1134	This work
FeNi/Ni-foam	350	/	4	75	99	/	7
Ni-$Fe_{0.25}$-Al/Ni-foam	231	/	4	67.5	99	/	8
Ni-SiO_2/GO/Ni-foam	470	500	4	83.7	91.0	3.06	9
Ni-Al_2O_3/Ni-foam	370	2500	4	94	99	18.70	10
RuNi/Ni-foam	300	/	4	73	99	0.00	11
NiPs/Ni-foam	450	/	4	70.4	95.5	0.00	12
10 wt% Ni-Al_2O_3	360	6000	4	83	98	39.22	13
27 wt% Ni/MgO	375	10,000	4	91.2	99	72.55	14
20 wt% Ni/TiO_2	400	48,000	4	52	100	200.57	15
Ni/ZrO_2	350	60,000	4	79.1-	100	0.00	16
12Ni4.5Ce/CNT	350	30,200	4	83.8	100	203.36	17
Ni/1MgO/SiO_2	350	15,000	4	67	98	79.14	18
Ni-Co/MCM-41	380	9000	4	85.6	99.8	61.78	19
15wt%Co/Al_2O_3	400	16,000	4	82	80	84.34	20
Co NRs	325	18,000	4	80	98	113.40	21
Ni-Co/CeO_2-ZrO_2	300	12,000	4	61	97	57.06	22
1.71Mn-Ni/Al_2O_3	450	48,000	4	80	99	305.49	23
15%Ni-5%Fe/AC	430	60,000	4	77	98	363.83	24
Ni-2%wtMn/Bn	270	3600	4	85.2	100	24.65	25
1Ru-15Ni/Al_2O_3	400	5835	5	88	82	33.83	26
Co_2C/γ-Al_2O_3	300	60,000	4	89	88	377.61	27
Ni/SiO_2-C	310	10,000	4	77.2	99.8	61.91	28
Ni/SiO_2-AEM	370	10,000	4	80	95	61.07	29
20Ni0.5Ru/Al_2O_3	350	40,000	4	82	100	263.57	30

Supplementary References

1. Jiang Feng, Wang Shanshan, Liu Bing, Liu Jie, Wang Li, Xiao Yang, Xu Yuebing, Liu Xiaohao. Insights into the Influence of CeO_2 Crystal Facet on CO_2 Hydrogenation to Methanol over Pd/CeO_2 Catalysts. *ACS Catal.* **10**, 11493-11509 (2020).

2. Xie Yu, Chen Jianjun, Wu Xi, Wen Junjie, Zhao Ru, Li Zonglin, Tian Guocai, Zhang Qiulin, Ning Ping, Hao Jiming. Frustrated Lewis Pairs Boosting Low-Temperature CO_2 Methanation Performance over Ni/CeO_2 Nanocatalysts. *ACS Catal.* **12**, 10587-10602 (2022).

3. Dou Liguang, Fu Mingkai, Gao Yuan, Wang Lei, Yan Cunji, Ma Tianzeng, Zhang Qiangqiang, Li Xin. Efficient sulfur resistance of Fe, La and Ce doped hierarchically structured catalysts for low-temperature methanation integrated with electric internal heating. *Fuel* **283**, 118984 (2021).

4. Gao Yuan, Dou Liguang, Zhang Shuai, Zong Lijun, Pan Jie, Hu Xiucui, Sun Hao, Ostrikov Kostya, Shao Tao. Coupling bimetallic Ni-Fe catalysts and nanosecond pulsed plasma for synergistic low-temperature CO_2 methanation. *Chem. Eng. J.* **420**, 127693 (2021).

5. Ma Haibin, Ma Kui, Ji Junyi, Tang Siyang, Liu Changjun, Jiang Wei, Yue Hairong, Liang Bin. Graphene intercalated Ni-SiO_2/GO-Ni-foam catalyst with enhanced reactivity and heat-transfer for CO_2 methanation. *Chem. Eng. Sci.* **194**, 10-21 (2019).

6. Li Yakun, Zhang Qiaofei, Chai Ruijuan, Zhao Guofeng, Cao Fahai, Liu Ye, Lu Yong. Metal-foam-structured Ni–Al_2O_3 catalysts: Wet chemical etching preparation and syngas methanation performance. *Appl. Catal., A* **510**, 216-226 (2016).

7. Dou Liguang, Yan Cunji, Zhong Liangshu, Zhang Dong, Zhang Jingye, Li Xin, Xiao Liye. Enhancing CO_2 methanation over a metal foam structured catalyst by electric internal heating. *Chem. Commun.* **56**, 205-208 (2020).

8. Chen Yaqi, Wu Xiaoren, Liu Qing, He Maoshuai, Bai Hongcun. Ni-Foam Structured Ni-Phyllosilicate Ensemble as an Efficient Monolithic Catalyst for CO_2 Methanation. *Catal. Lett.* **152**, 2738-2744 (2022).

9. Jaffar Mohammad M., Nahil Mohamad A., Williams Paul T. Parametric Study of CO_2 Methanation for Synthetic Natural Gas Production. *Energy Technol.* **7**, 1900795 (2019).

10. Loder A., Siebenhofer M., Lux S. The reaction kinetics of CO_2 methanation on a bifunctional Ni/MgO catalyst. *J. Ind. Eng. Chem.* **85**, 196-207 (2020).

11. Unwiset Preeya, Chanapattharapol Kingkaew Chayakul, Kidkhunthod Pinit, Poo-arporn Yingyot, Ohtani Bunsho. Catalytic activities of titania-supported nickel for carbon-dioxide methanation. *Chem. Eng. Sci.* **228**, 115955 (2020).

12. Jia Xinyu, Zhang Xiaoshan, Rui Ning, Hu Xue, Liu Chang-jun. Structural effect of Ni/ZrO_2 catalyst on CO_2 methanation with enhanced activity. *Appl. Catal., B* **244**, 159-169 (2019).

13. Wang Wei, Chu Wei, Wang Ning, Yang Wen, Jiang Chengfa. Mesoporous nickel catalyst supported on multi-walled carbon nanotubes for carbon dioxide methanation. *Int. J. Hydrogen Energy* **41**, 967-975 (2016).

14. Guo Meng, Lu Gongxuan. The effect of impregnation strategy on structural characters and CO_2 methanation properties over MgO modified Ni/SiO_2 catalysts. *Catal. Commun.* **54**, 55-60 (2014).

15. Wang Xiaoliu, Zhu Lingjun, Liu Yincong, Wang Shurong. CO_2 methanation on the catalyst of Ni/MCM-41 promoted with CeO_2. *Sci. Total Environ.* **625**, 686-695 (2018).

16. Liang Chuanfei, Tian Hongli, Gao Guoming, Zhang Shu, Liu Qing, Dong Dehua, Hu Xun. Methanation of CO_2 over alumina supported nickel or cobalt catalysts: Effects of the coordination between metal and support on formation of the reaction intermediates. *Int. J. Hydrogen Energy* **45**, 531-543 (2020).

17. Jimenez Juan D., Wen Cun, Lauterbach Jochen. Design of highly active cobalt catalysts for CO_2 hydrogenation via the tailoring of surface orientation of nanostructures. *Catal. Sci. Technol.* **9**, 1970-1978 (2019).

18. Pastor-Pérez L., Patel V., Le Saché E., Reina T. R. CO_2 methanation in the presence of methane: Catalysts design and effect of methane concentration in the reaction mixture. *J. Energy Inst.* **93**, 415-424 (2020).

19. Zhao Kechao, Li Zhenhua, Bian Li. CO_2 methanation and co-methanation of CO and CO_2 over Mn-promoted Ni/Al_2O_3 catalysts. *Front. Chem. Sci. Eng.* **10**, 273-280 (2016).

20. Gonçalves Liliana P. L., Sousa Juliana P. S., Soares O. Salomé G. P., Bondarchuk Oleksandr, Lebedev Oleg I., Kolen'ko Yury V., Pereira M. Fernando R. The role of surface properties in CO_2 methanation over carbon-supported Ni catalysts and their promotion by Fe. *Catal. Sci. Technol.* **10**, 7217-7225 (2020).

21. Chein Rei-Yu, Wang Chih-Chang. Experimental Study on CO_2 Methanation over Ni/Al_2O_3, Ru/Al_2O_3, and Ru-Ni/Al_2O_3 Catalysts (2020).

22. Valinejad Moghaddam Shima, Rezaei Mehran, Meshkani Fereshteh, Daroughegi Reihaneh. Carbon dioxide methanation over Ni-M/Al_2O_3 (M: Fe, CO, Zr, La and Cu) catalysts synthesized using the one-pot sol-gel synthesis method. *Int. J. Hydrogen Energy* **43**, 16522-16533 (2018).

23. Ye Run-Ping, Liao Lin, Reina Tomas Ramirez, Liu Jiaxu, Chevella Durgaiah, Jin Yonggang, Fan Maohong, Liu Jian. Engineering Ni/SiO_2 catalysts for enhanced CO_2 methanation. *Fuel* **285**, 119151 (2021).

24. Ye Run-Ping, Gong Weibo, Sun Zhao, Sheng Qingtao, Shi Xiufeng, Wang Tongtong, Yao Yi, Razink Joshua J., Lin Ling, Zhou Zhangfeng, Adidharma Hertanto, Tang Jinke, Fan Maohong, Yao Yuan-Gen. Enhanced

stability of Ni/SiO_2 catalyst for CO_2 methanation: Derived from nickel phyllosilicate with strong metal-support interactions. *Energy* **188**, 116059 (2019).

25. Stangeland Kristian, Kalai Dori Yosef, Li Hailong, Yu Zhixin. Active and stable Ni based catalysts and processes for biogas upgrading: The effect of temperature and initial methane concentration on CO_2 methanation. *Appl. Energy* **227**, 206-212 (2018).

26. Rahmani Soudabeh, Rezaei Mehran, Meshkani Fereshteh. Preparation of highly active nickel catalysts supported on mesoporous nanocrystalline γ-Al_2O_3 for CO_2 methanation. *J. Ind. Eng. Chem.* **20**, 1346-1352 (2014).
